# Supplementary material for: Biofilm Formation by Staphylococcus aureus Clinical Isolates is Differentially Affected by Glucose and Sodium Chloride Supplemented Culture Media
Source: J Clin Med. 2019 Nov 2;8(11):1853. doi: 10.3390/jcm8111853 (PMC6912320; doi:10.3390/jcm8111853)
Supplement: Supplementary file 1 [file jcm-08-01853-s001.pdf]

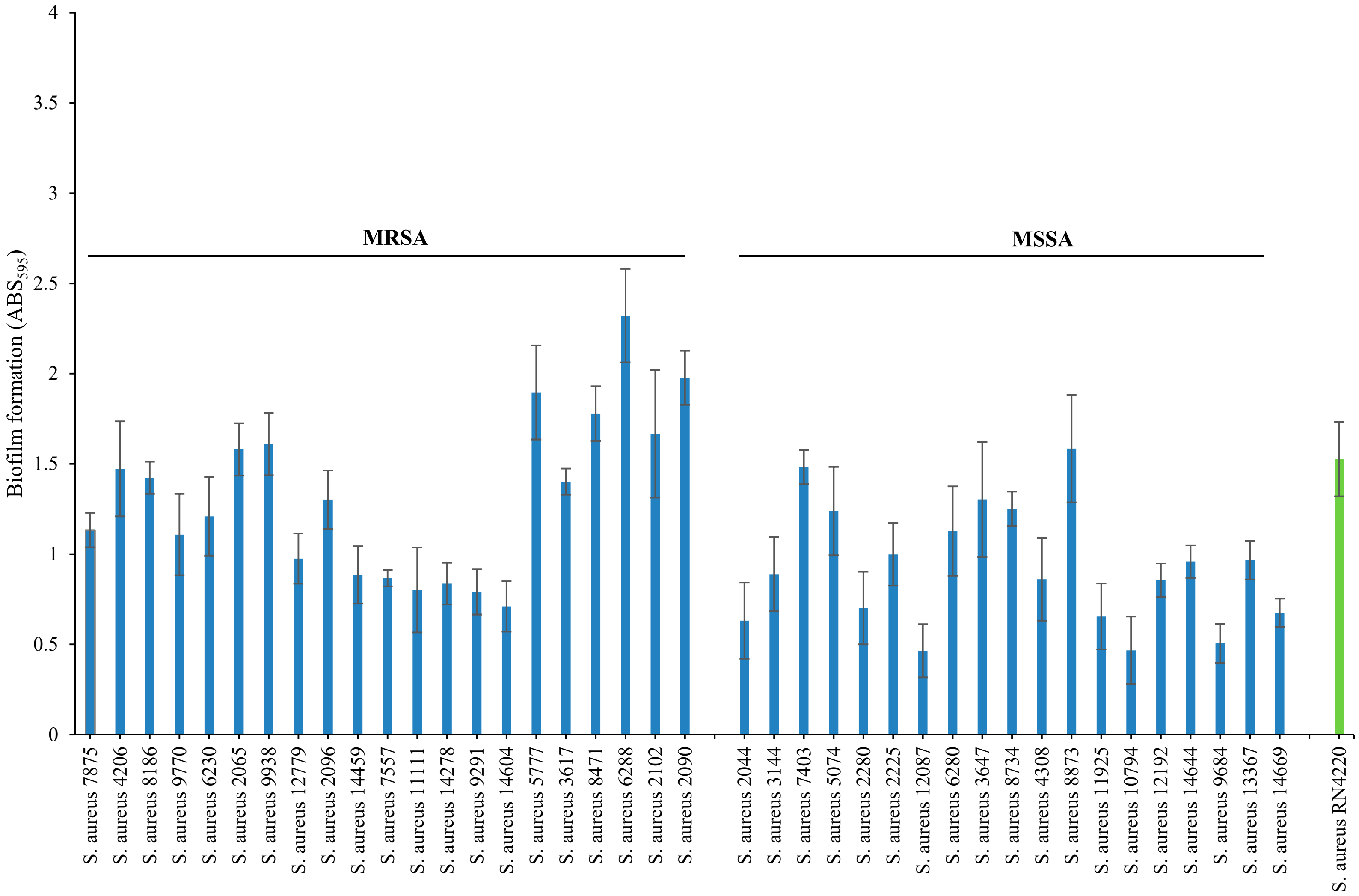

Figure S1. Biofilm formation (ABS_{595}) of MRSA and MSSA strains grown in TSB supplemented with 0.5% glucose. The green bar shows results for the positive control strain RN4220. The data show the mean, and error bars represent the standard deviation within the replicates, for at least two independent experiments

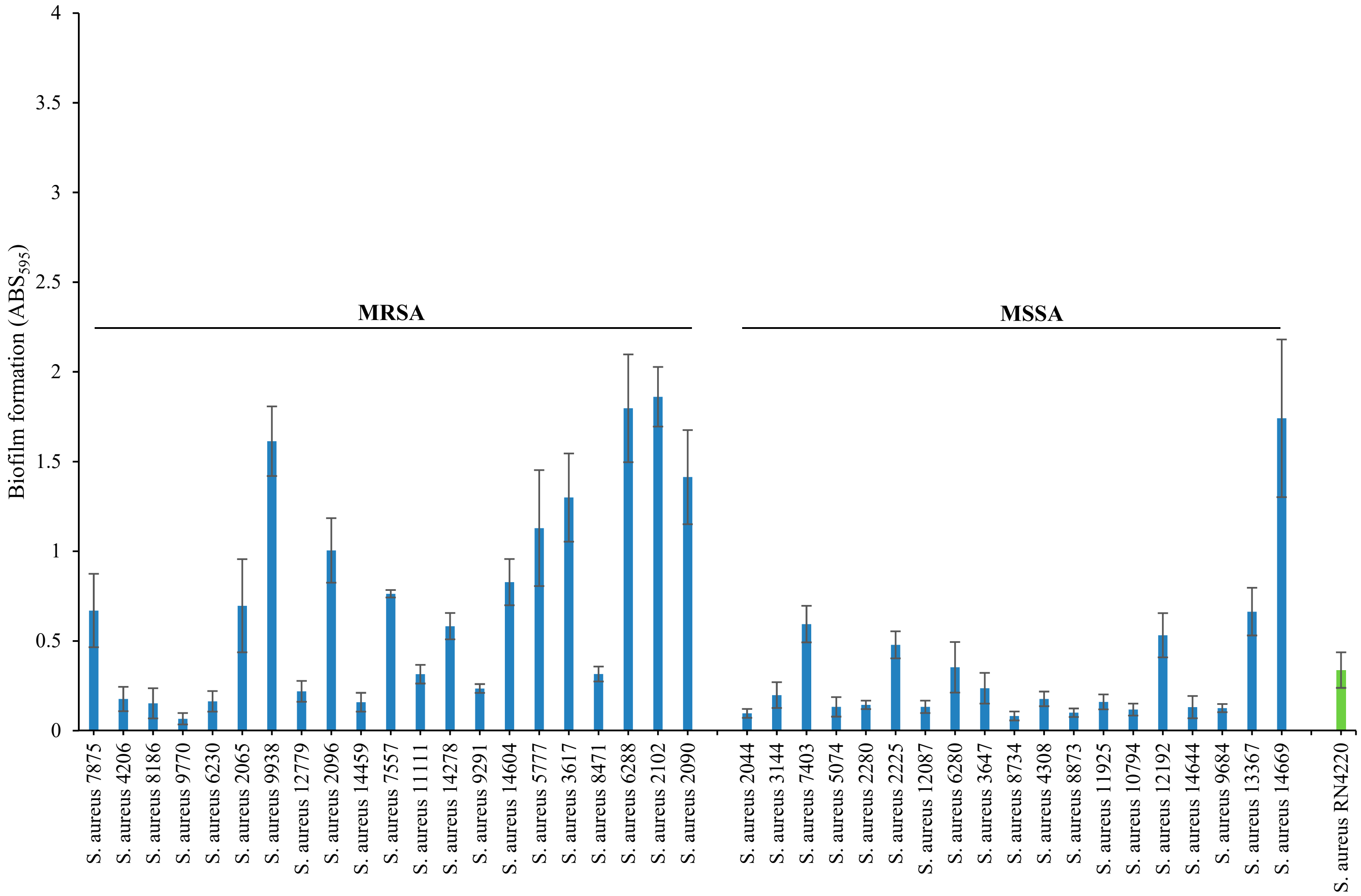

Figure S2. Biofilm formation (ABS_{595}) of MRSA and MSSA strains grown in TSB supplemented with 1.0% NaCl. The green bar shows results for the positive control strain RN4220. The data show the mean, and error bars represent the standard deviation within the replicates, for at least two independent experiments.

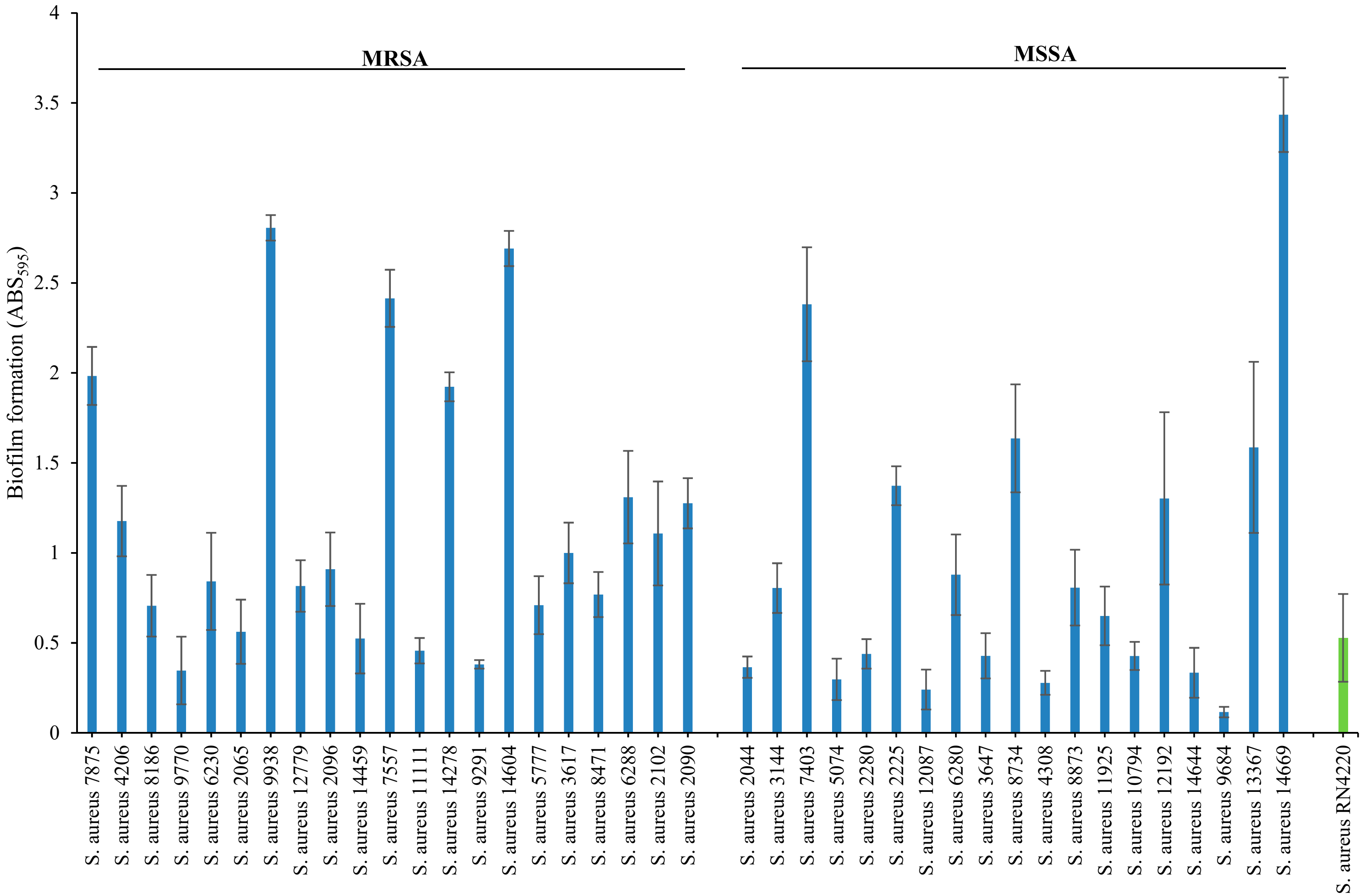

Figure S3. Biofilm formation (ABS_{595}) of MRSA and MSSA strains grown in TSB supplemented with 2.0% NaCl. The green bar shows results for the positive control strain RN4220. The data show the mean, and error bars represent the standard deviation within the replicates, for at least two independent experiments.

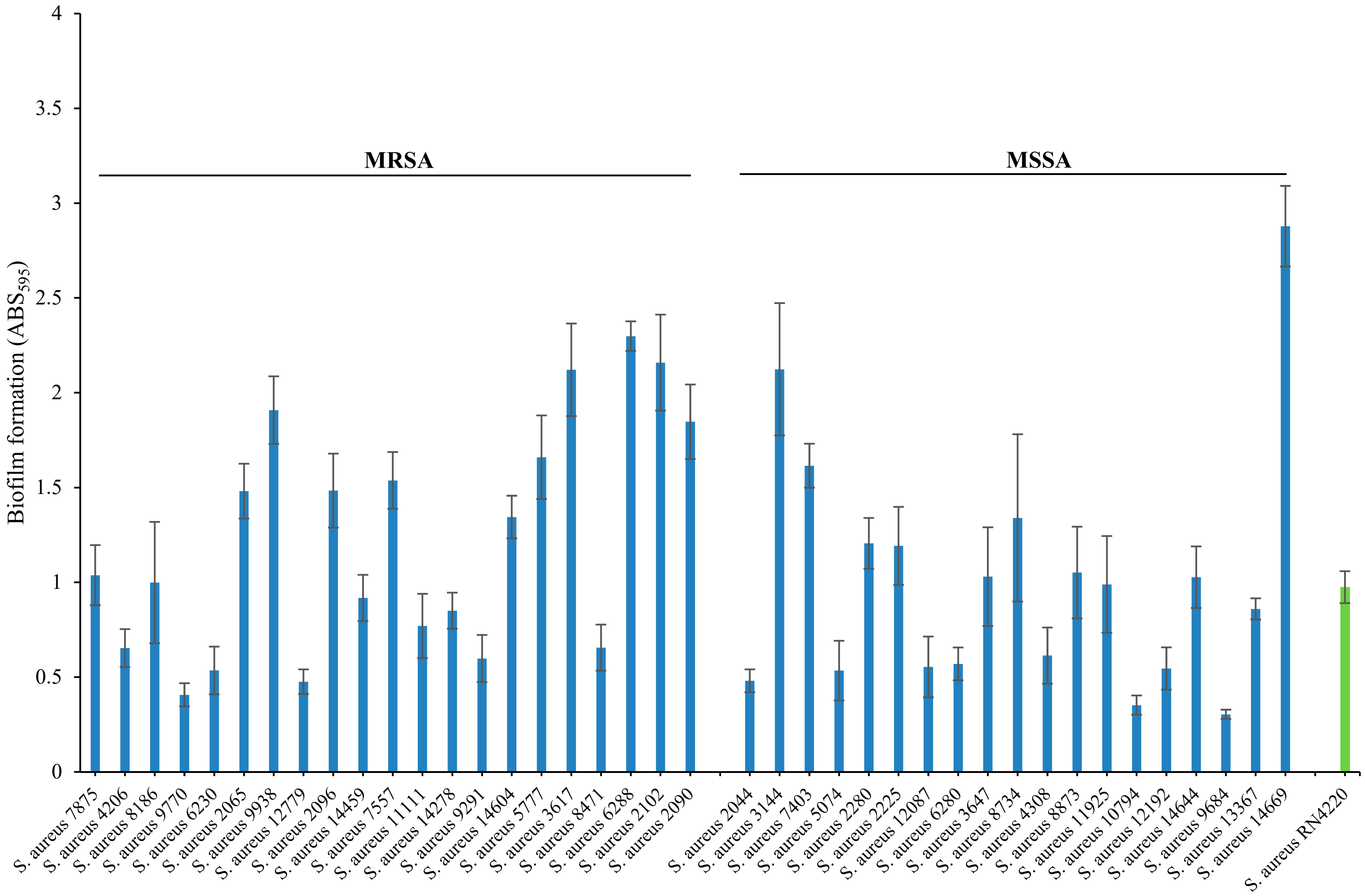

Figure S4. Biofilm formation (ABS_{595}) of MRSA and MSSA strains grown in TSB supplemented with 1.0% glucose and 1.0% NaCl. The green bar shows results for the positive control strain RN4220. The data show the mean, and error bars represent the standard deviation within the replicates, for at least two independent experiments.
